# An International Virtual Classroom: The Emergency Department Experience at Weill Cornell Medicine and Weill Bugando Medical Center in Tanzania

**DOI:** 10.9745/GHSP-D-21-00005

**Published:** 2021-09-30

**Authors:** Lynn G. Jiang, Peter W. Greenwald, Michael J. Alfonzo, Jane Torres-Lavoro, Manish Garg, Ally Munir Akrabi, Erasto Sylvanus, Shahzmah Suleman, Radhika Sundararajan

**Affiliations:** a Department of Emergency Medicine, New York-Presbyterian Weill Cornell Medical Center, New York, NY.; b Division of Pediatric Emergency Medicine, Department of Emergency Medicine, New York-Presbyterian Weill Cornell Medical Center, New York, NY.; c Department of Emergency Medicine, Weill Bugando Medical Center, Mwanza, Tanzania.; d Center for Global Health, Weill Cornell Medicine, New York, NY.

## Abstract

Emergency medicine (EM) is rapidly being recognized as a specialty around the globe. This has particular promise for low- and middle-income countries (LMICs) that experience the largest burden of disease for emergency conditions. Specialty education and training in EM remain essentially an apprenticeship model. Finding the required expertise to educate graduate learners can be challenging in regions where there are low densities of specialty providers.

We describe an initiative to implement a sustainable, bidirectional partnership between the Emergency Medicine Departments of Weill Cornell Medicine (WCM) in New York, NY, USA, and Bugando Medical Center (BMC) in Mwanza, Tanzania. We used synchronous and asynchronous telecommunication technology to enhance an ongoing emergency medicine education collaboration.

The Internet infrastructure for this collaboration was created by bolstering 4G services available in Mwanza, Tanzania. By maximizing the 4G signal, sufficient bandwidth could be created to allow for live 2-way audio/video communication. Using synchronous and asynchronous applications such as Zoom and WhatsApp, providers at WCM and BMC can attend real-time didactic lectures, participate in discussion forums on clinical topics, and collaborate on the development of clinical protocols. Proof of concept exercises demonstrated that this system can be used for real-time mentoring in EKG interpretation and ultrasound technique, for example. This system was also used to share information and develop operations flows during the COVID-19 pandemic. The use of telecommunication technology and e-learning in a format that promotes long-term, sustainable interaction is practical and innovative, provides benefit to all partners, and should be considered as a mechanism by which global partnerships can assist with training in emergency medicine in LMICs.

## Abstract (precis)

We created a sustainable, bidirectional partnership using telecommunication technology to enhance emergency medicine education collaboration. Telemedicine is a practical and innovative methodology to expand training in emergency medicine and establish bidirectional partnerships between academic departments in high-income and low- and middle-income countries.

## Key Messages

- Specialty graduate medical education and training typically follows an apprenticeship model that requires in-person teaching and instruction, which because of the COVID-19 pandemic, can be challenging in regions with travel restrictions and strict lockdown guidelines. In areas that have few specialty-trained local providers, it presents a circular conundrum: creating training programs requires faculty; having faculty requires creating training programs.
- The bidirectional partnership established between the emergency medicine departments of Weill Cornell Medicine and Bugando Medical Center demonstrates a collaborative approach to telemedicine and e-learning that is sustainable and encourages long-term engagement. Our approach of incorporating telecommunications into academic collaborations may remediate problems that have reduced the effectiveness of other traditional forms of educational connection between high-income countries and low- and middle-income countries, including lack of sustainability and reduced engagement over time.
- Although this technology was used in the context of emergency medicine graduate education, the benefits of this approach can easily be extrapolated to strengthen a wide variety of international collaborations.

## EMERGENCY MEDICINE AS A GROWING SPECIALTY

In the United States, emergency medicine (EM) was recognized as a specialty in 1979.^1^ Since that time the specialty has developed rapidly; now, more than 30 countries formally recognize the specialty. In countries where EM is new, residency training programs and certifications in EM are being developed.^2^ The expansion of this specialty has particular promise for low and middle-income countries (LMICs), as they bear a disproportionate burden of emergency conditions compared to high-income countries (HICs).^3^

In sub-Saharan Africa, in particular, it has been shown that starting EM training programs can dramatically reduce hospital-wide mortality.^4^^–^^6^

Specialty graduate medical education and training typically follows an apprenticeship model, where the principles of clinical care are acquired through longitudinal contact over years with experienced specialists who provide instruction grounded in the clinical realities of their specialty.^8^^,^^9^ This often requires in-person teaching and instruction, which can be challenging in regions where there are low densities of specialty providers. Building graduate programs where there are few specialty-trained local providers presents a circular conundrum: to create the required training programs, faculty are needed; to have faculty, training programs must be created.

In Africa, medical education partnerships between HICs and local institutions have tended to focus on creating educational infrastructure. This has corresponded to increased support for undergraduate medical education in sub-Saharan Africa and a concomitant increase in medical school capacity.^10^^,^^11^ Despite efforts to expand public health and research capacity with the help of international funding, support for the specialty training after medical school has not developed at the same pace.^12^^,^^13^ Lack of clinical mentorship and educational support have been identified as critical limitations to specialty education in sub-Saharan Africa.^14^^,^^15^

Lack of clinical mentorship and educational support have been identified as critical limitations to specialty education in sub-Saharan Africa.

EM education opportunities in Tanzania are currently limited, and when present, they are in the early stages of development. In Tanzania, the first and only EM residency program was established in 2010. The nation of Tanzania has a population of 44 million people and a total of 35 EM-trained specialists.^7^ The majority of emergency departments (EDs) are staffed by providers with little to no formal EM training so potential trainees often have to look for positions in other countries for formal training.^7^ Development of further residency programs would require “buy-in” and regulatory approval from the national government which has not occurred to date. In the context of these limitations, we hope that our collaborative efforts can help support the efforts of the EM-trained Bugando Medical Center (BMC) staff in Mwanza, Tanzania, to build EM knowledge among local providers.

Much of the current academic literature on this topic, particularly those that examine clinically oriented partnerships between the United States and African medical centers, indicates that there have been ongoing issues with maintaining and sustaining connection. Several studies discuss the subsequent lack of consistent training for African providers and overall inconsistent U.S. partnership presence for African programs.^16^^,^^17^ Although programs such as the Medical Education Partnership Initiative have attempted to address the disparities, there are still many examples where partnerships between HICs and LMICs are less beneficial or even harmful.^18^^–^^20^ Electronic learning (e-learning) and other types of remote education have gained new prominence in the setting of the coronavirus disease (COVID-19) pandemic.^21^^,^^22^ It may be that some of these same tools, coupled with partnerships between specialty faculty in HICs and LMICs, may be a method to address this lack of clinical mentorship and support.

## USING E-LEARNING TO PROMOTE EM TRAINING

E-learning platforms have previously been proposed as a mechanism to expand access to medical education in sub-Saharan Africa.^23^^–^^25^ In theory, e-learning can allow self-directed education that mitigates the limited availability of specialists.^24^^,^^25^ However, in many cases, e-learning programs have not lived up to expectations and have been criticized for poor organization and misalignment with local conditions and priorities.^24^ Barriers to effective e-learning have included difficulties associated with obtaining the required electronic resources, integrating curriculum, and motivating participants to initiate and sustain their engagement.^26^^–^^28^ It may be possible to address some of these e-learning shortcomings in the context of HIC/LMIC medical partnerships by using electronic communications media to foster the same relationships and longitudinal connection that occurs with traditional face-to-face instruction. This same ongoing engagement could also be dynamically structured to help ensure that educational content remains aligned with local needs and priorities.

Barriers to effective e-learning have included difficulties associated with obtaining the required electronic resources, integrating curriculum, and motivating participants to initiate and sustain their engagement.

The timing may be opportune for telecommunication use in global specialty medical educational partnerships. In the United States, as well as in many other parts of the world, telemedicine care delivery systems, including provider-to-provider consultations and direct patient care, have become part of mainstream practice.^29^^–^^31^ As this type of care grows, providers and graduate medical educators become more comfortable with telecommunications technology and the use of this technology in education increases.

EM at Weill Cornell Medicine (WCM) in New York is an example of an academic department where the delivery of telemedicine care and remote e-learning have developed together.^32^^–^^34^ In this context, we felt it would be of interest to the greater community to share the way we have used these telecommunication technologies to support the collaborative relationship between the EDs of WCM and BMC. Here, we describe our initiative to implement a sustainable, bidirectional partnership using both synchronous and asynchronous telecommunication to support EM training and use this technology to enhance traditional models of clinical education. We hope that this work may suggest a useful avenue for other global partnerships.

## WCM AND BMC EM DEPARTMENT COLLABORATION

WCM and BMC have a decades-long history of cooperation beginning in the 1980s (Box).^35^ In recent years, both institutions have seen rapid development within their EM programs with BMC creating a formal ED in 2017 and WCM establishing an academic Department of EM in 2018.^36^^,^^37^

BOX **Background of Weill Cornell Medicine and Bugando Medical Center Collaboration**

WCM was established as a medical school in 1898 and has been affiliated with New York- Presbyterian Hospital since 1913.^37^ A formal residency for EM training was established at New York-Presbyterian Hospital and WCM in 2003, and the division of EM transitioned to the Department of EM in 2018. The Department has over 80 EM board-certified faculty caring for 150,000 patients a year and is home to the WCM Center for Virtual Care, a center chartered to promote development and education in telemedicine.^38^

BMC was built by the Catholic Church and opened in 1971. In 2003, the Catholic University College of Health and Allied Sciences (CUHAS) opened the first medical college in western Tanzania at the same location. WCM established a formal affiliation with CUHAS and BMC in 2003. Since its establishment, over 1,000 Tanzanian physicians have graduated from CUHAS. BMC is the zonal referral hospital for the Lake Zone of Tanzania, with a catchment population of 18 million people. The ED has an annual volume of 35,000 patients with varied acuity. The BMC ED is staffed by 3 EM specialist physicians supervising 33 entry-level physicians and 22 nurses in clinical care.^36^

In 2018, a collaboration between BMC and WCM EM departments began with a bidirectional exchange of personnel for both clinical electives and research. In addition, a 2-month standard rotation at BMC was developed for WCM Pediatric EM fellows. In 2019, 2 BMC EM specialists participated in a 1-month observership at WCM, and a 4-week elective at BMC was established for senior EM WCM residents.

While rotating at BMC, WCM residents and fellows participated in oversight of patient care and deliver lectures on common clinical presentations. Through these educational opportunities, WCM and BMC have jointly developed department protocols to streamline patient care for common chief complaints.

Through bidirectional exchange opportunities, WCM and BMC have jointly developed department protocols to streamline patient care for common chief complaints.

### Developing the Internet Infrastructure to Promote Educational Exchange

In fall 2019, BMC and WCM EDs jointly decided to strengthen and establish systematic interactions between the departments by developing Internet capacity that would allow for more regular educational exchange. It was felt that building this technology capacity would allow for organic development of educational programming. A stakeholder assessment at BMC indicated that Internet infrastructure was limited; total Internet for the institution, including clinical services for the 900-bed hospital, had a bandwidth of 10Mbps, and this limited bandwidth was subject to frequent service outages. In addition, materials needed to facilitate telecommunication, such as laptops and webcams, were not widely available.

To increase Internet capacity, an “always-on” high-speed Internet connection was considered the preferred solution. A cabled connection was not available in the region. As an alternative, BMC faculty suggested using the existing 4G cellular service. This led to the development of a system (detailed below) that included a 4G antenna mounted on the roof, a cable connection to the ED’s ground floor location, and a cell signal repeater, as well as a multiband 4G router to achieve 24 Mbps download and 15 Mbps upload speed.

Building and maintaining Internet capacity was paid for using departmental funds from WCM EM. Equipment included a wireless router device that uses a SIM card, the same type of SIM card used in a cellular phone. Trials of several wireless service providers in Mwanza indicated that the fastest service was available from the local Tanzanian Vodacom Company. The SIM card solution provided relatively inexpensive Internet, albeit with the drawback that the available data is “capped” (after a given amount of data is transmitted, the card must be “recharged”). Comparison of readily available “hotspot” SIM card modems sold by the same company with a multiband SIM card router (a router that makes use of signals from more than 1 cell tower and more than 1 frequency at a time) indicated faster connection speed with the multiband router. A rooftop antenna, coaxial cable, and a multiband 4G signal repeater, along with the multiband SIM card router, were purchased from eBay and Amazon in the United States, where the equipment was less costly. The resulting connection allowed 24 Mbps download speed, 15 Mbps upload speed, and a latency of 30–40 milliseconds. In addition to the connection equipment, funding was also made available for 2 laptop computers, a camera, and microphone equipment to allow for video conferencing through the Zoom application. The (Table) indicates initial start-up costs of equipment in addition to ongoing costs.

**TABLE.** Telecommunication Equipment and Costs

**Equipment**	**Description**	**Cost, US$**
Two Hewlett Packard 15.6" laptops	Computers	1,062.62
Indmire laptop battery for Hewlett Packard Pavilion	Extra laptop battery	42.49
Logitech high-definition pro webcam	Webcam	60.97
Two Owl conference room cameras	Voice activated, swiveling cameras (one for BMC, one at WCM) (purchased refurbished)	1,398.00
Innogear upgraded adjustable microphone stand	Desktop Microphone Stand	12.99
UHF wireless microphone	USB Microphone/UHF microphone with USB receiver for laptop	31.99
Two Lysignal outdoor/indoor wall mount directional panel antenna 698 to 2700 MHz	Antennas to distribute 4G signal in department	39.98
OB directional log-periodic antenna	Antenna able to receive 4G signal on roof (purchased used)	50.00
Cellphone signal booster	Cell signal repeater kit, to boost 4G signal within Department after signal delivered to ground floor by cable.	287.78
Teltonika RUT950 mobile 4G/LTE dual SIM slot modem with 802.11n wireless router	Multiband sim card wireless router	214.98
Wilson Electronics 75 ft. coax cable	Cable to connect ground floor to roof	62.64
Wilson Electronics 55 ft. coax cable	Cable to connect signal booster to indoor antennas	53.77
Ancable N-Type male to dual 2 N Type female 3-way splitter	Cable connection material	11.99
Ancable 2-pack premium N type ohm barrels adaptor coupler	Connectors for cell signal booster	8.99
**Total set-up cost**		3,339.19
Bandwidth expense	Recurring Sim recharge for 90GB of data (approximate)	approx. 40/month

Abbreviations: BMC, Bugando Medical Centre; USB, universal serial bus; WCM, Weill Cornell Medicine.

### Establishing a Collaborative Educational Exchange

We intended to build adequate Internet capacity first, then mutually determine what types of collaboration made the most sense for the 2 departments; this remains an iterative process. To date, we have used synchronous and asynchronous software platforms to support joint conferences, lectures, and the sharing of medical education resources.

WCM EM faculty provide real-time Zoom lectures on a variety of topics, including EKG interpretation, toxicology, pediatric drowning, and ultrasound instruction. These lectures occur once a month (late night United States time, morning East Africa time). With BMC faculty input, these sessions are designed for the needs of clinical learners in Mwanza. Based on this guidance, these lectures emphasize the development of clinical protocols and systematic care approaches. To allow for off-hours exchange of educational materials, WCM EM didactic lectures on clinically relevant topics including burn care, facial trauma, pediatric drowning, and chest pain have been recorded and placed on a shared hard drive to be viewed at individuals’ convenience. BMC staff have also attended the weekly WCM EM didactic conferences in real-time via Zoom. Additionally, we performed “proof of concept” interactions where U.S.-based faculty members guided BMC providers in real-time ultrasound acquisition.

WCM EM faculty provide real-time Zoom lectures on a variety of topics, including EKG interpretation, toxicology, pediatric drowning, and ultrasound instruction.

We have also established WhatsApp messaging groups between WCM and BMC clinicians that support asynchronous questions and feedback on clinical topics. For example, a WhatsApp group dedicated to EKG interpretation, where de-identified EKGs are posted by BMC ED clinicians. All members of the group can respond with guidance on interpretation or pose additional clinical questions. Other WhatsApp messaging groups also allow for discussing, scheduling, providing updates on departmental news, and supporting real-time troubleshooting.

We continue to develop and expand our implementation of this technology to provide further clinical support. Additional collaborations include the Helping Children Survive initiative to train both BMC ED staff as well as BMC Department of Pediatrics staff in Pediatric Emergency Assessment Recognition and Stabilization that is adapted to a resource-limited setting. BMC has also incorporated a structured note template for pediatric admissions to create a local clinical information network. Research collaboration is ongoing on topics such as the evaluation and impact of delayed referrals for trauma patients and the examination of ED point-of-care ultrasound to improve outcomes among patients with heart failure. When not directly present with one another, these research teams maintain communication through WhatsApp, email, and Zoom meetings.

BMC faculty conduct real-time (afternoon East Africa time, morning New York time) Zoom lectures to WCM learners on clinical cases in Mwanza that involve pathologies and presentations that are uncommon in HIC settings, and therefore educationally valuable for the WCM residents. The clinical cases are incorporated into the WCM EM residency curriculum. These joint conferences, conducted monthly, explore clinical concepts relevant to both departments with shared presentations and discussion of teaching points to illustrate the practice of EM in different clinical environments.

In March 2020, given that the pandemic epicenter was currently in New York City and estimated to peak in eastern Africa in the coming months, colleagues from BMC sought clinical operations guidance to prepare themselves for a COVID-19 outbreak in western Tanzania. The WCM ED clinical operations team conducted a 90-minute Zoom conference for BMC colleagues describing triage, screening procedures, and infection control mechanisms in clinical areas. In June 2020, the BMC ED team presented via Zoom their experiences of establishing and caring for patients in their ED-based COVID-unit. Many of their clinical protocols were developed based on WCM experiences from the surge earlier that year. Tanzania has subsequently experienced a second resurgence of COVID cases that has limited WCM’s ability to send personnel to Tanzania. However, our established telecommunication channels and sustained relationship have enabled us to offer continued educational and clinical support in the form of Zoom lectures and clinical guidance.

## DISCUSSION

Traditional global health EM collaborations have often focused on short-term, in-person experiences, sending individuals from HICs to LMICs for a time-limited rotation or to oversee project implementation. Although sustainability is increasingly a central criterion in global health initiatives, resources are not always directed toward infrastructure to maintain these efforts beyond the individuals’ physical presence.^39^^,^^40^ Frustration with such discontinuities can lead to a disproportionate benefit to HICs while leading to LMIC participant disillusionment, posing obstacles to future collaborative relationships.^41^^,^^42^ The long-term impact and benefits of global EM collaborations depend on building and maintaining partner involvement through ongoing, bidirectional interaction. We described a telecommunications approach that has the potential to mitigate discontinuity and be a model for remote academic partnerships between international EDs. Although there were start-up costs and effort required to establish the necessary technologic infrastructure, these investments in money and effort are relatively small compared to the expense and time required for round-trip travel between Mwanza and New York City.

The long-term impact and benefits of global EM collaborations depend on building and maintaining partner involvement through ongoing, bidirectional interaction.

Even before the onset of the COVID-19 pandemic, we felt that the increasing familiarity with electronic communication might make the time right to explore the use of these tools as part of our international collaboration. Events associated with the pandemic have strengthened our conviction on this point.^43^ The impact of the pandemic has demonstrated that global mobility is fragile. Travel restrictions and quarantine guidelines underscore the importance of establishing systems for partnerships that can be maintained outside of recurrent physical presence. Telemedicine technology allows us to continue specialty medical education initiatives even at long distances. The use of this technology in the context of EM graduate education has become common, and its benefits can easily be extrapolated to strengthen a wide variety of international collaborations.^44^ A combination of asynchronous and real-time communication also alleviates the numerous shortcomings that have been described for e-learning as a sustainable approach to global education.^24^^,^^26^ The increasing availability of high-speed Internet in many areas of the globe, coupled with the prospect of increased Internet availability that may occur following the deployment of low earth orbit satellite networks, will continue to reduce the technical obstacles to this type of collaboration.^45^

### Limitations

There are some limitations to this approach. Synchronous interactions can be challenging to schedule due to the time difference between our sites (7 or 8 hours, depending on U.S. Daylight Savings Time). Scheduling lectures at different mutually agreed upon times rather than 1 recurrent time means that both partners equally bear potential inconveniences, but the fact remains that the time difference creates inconveniences for both parties. One potential advantage to the time difference is that it allows individuals with different clinical schedules—like nocturnists—an opportunity to participate. Asynchronous techniques like the WhatsApp groups and the recorded lectures have also been useful in overcoming time difference obstacles.

While we report preliminary anecdotal success with this initiative, further research is needed to assess the effectiveness of the approach in global graduate medical education. We are in the process of obtaining formal feedback for joint conferences and lectures in reaching educational graduate medical education milestones for both WCM and BMC participants.^46^^,^^47^ In the meantime, we plan to expand our collaborative projects through the creation of a WhatsApp toxicology consult group, allowing for asynchronous discussion of toxicology principles. Our ultimate goal is to use this technology as a mechanism to support the establishment of an EM residency program at BMC while simultaneously broadening the exposure of US residents to pathology not frequently encountered during their training.

Although we have used this technology specifically for education, there is also potential to develop these telecommunication tools to support virtual clinical teaching in the direct care of patients. Currently, however, there are only preliminary regulations regarding the provision of remote clinical care across international borders. These remain broad without the necessary specificities to the local health care environment. Without these, questions regarding legal and ethical guidelines to providing direct patient care in other international health care systems remain unanswered and limit the clinical support we can provide.

There is potential to develop these telecommunication tools to support virtual clinical teaching in the direct care of patients.

## CONCLUSION

Telemedicine is a practical and innovative way to expand specialty training in EM in LMICs and establish bidirectional partnerships between HIC and LMIC academic departments. Our approach of incorporating telecommunication into academic collaborations could remediate problems that have reduced the effectiveness of e-learning and other traditional forms of educational connection between HIC and LMIC, including lack of sustainability and limited engagement over time. We seek to implement a working relationship between distant partners that more closely approximates real-time, long-term in-person interactions. Integrating modalities of telemedicine into a learning environment aligns the experience with other proven graduate medical educational methodologies.

## Notes

**Peer Reviewed**

**First published online:** August 24, 2021.
